# Exercise as an adjunct intervention for social anxiety disorder: a mini-review

**DOI:** 10.3389/fpsyg.2026.1894573

**Published:** 2026-07-22

**Authors:** Liu Lele, Yang Xin, Yong-Gwan Song

**Affiliations:** 1 Pukyong National University, Busan, Republic of Korea; 2 Zhengzhou College of Finance and Economics, Zhengzhou, China

**Keywords:** adjunctive intervention, cognitive-behavioral therapy, exercise, physical activity, psychosocial functioning, social anxiety disorder

## Abstract

Social anxiety disorder (SAD) is a common and often persistent mental health condition that typically emerges during adolescence and is associated with marked functional impairment. Although cognitive-behavioral therapy and selective serotonin reuptake inhibitors remain first-line treatments, many individuals experience incomplete response, poor tolerability, stigma-related concerns, or limited access to care. Against this background, exercise has attracted increasing interest as a low-cost, accessible, and scalable adjunctive intervention, although adherence and accessibility may vary across populations. In this mini-review, we synthesize current evidence on the role of physical activity and structured exercise in the management of SAD. Available findings suggest that higher levels of physical activity are generally associated with lower social anxiety symptoms, while emerging intervention studies indicate that aerobic exercise, climbing, and group-based exercise programs may confer psychological benefit. Exercise may complement mainstream therapies through partially overlapping yet distinct pathways, including enhancement of self-efficacy, resilience, body image, and social engagement, as well as modulation of cognitive bias, neuroplasticity, and stress-related physiological processes. Potential applications in adolescents, individuals with autism spectrum disorder and comorbid social anxiety, and performance-related anxiety contexts also warrant attention. However, the current evidence base remains limited by small samples, heterogeneous interventions, insufficient long-term follow-up, and a lack of large randomized controlled trials in clinically diagnosed SAD, and much of the available evidence derives from non-clinical or subclinical populations. Overall, exercise should be considered a promising adjunct rather than a replacement for established treatments, and future research should prioritize mechanism-informed, precision-based, and implementation-oriented intervention models.

## 1 Introduction

Social anxiety disorder (SAD) is a prevalent psychiatric condition characterized by marked and persistent fear of social situations. Epidemiological estimates indicate a lifetime prevalence of approximately 4–13% across populations, placing it among the most common anxiety disorders and a leading psychiatric contributor to years lived with disability worldwide. Typically emerging during adolescence, untreated SAD frequently follows a chronic course, producing substantial impairment of personal functioning and considerable socioeconomic burden (GBD 2019 Mental Disorders Collaborators, n.d.; Szuhany and Simon, 2022; Aune et al., 2022). Although cognitive-behavioral therapy (CBT) and selective serotonin reuptake inhibitors (SSRIs) have been established as first-line treatments, a substantial proportion of patients exhibit inadequate therapeutic responses or cannot tolerate pharmacological side effects, reflecting a well-documented phenomenon of treatment resistance (Domschke et al., 2024b; Domschke et al., 2024a). Additional barriers, including stigma associated with psychiatric medication, avoidance of face-to-face psychotherapy, and limited treatment accessibility, further constrain the widespread implementation of conventional interventions (Hoge et al., 2020).

Against this backdrop, identifying accessible, generally well-tolerated, and readily integrable adjunctive or alternative strategies has emerged as a priority in clinical research. Regular physical activity and structured exercise have attracted growing interest owing to their broad physical and psychological health benefits. Accumulating evidence indicates that higher levels of physical activity correlate with reduced anxiety symptomatology (Herring et al., 2021; Zika and Becker, 2021), while targeted exercise training demonstrates clear potential for ameliorating symptoms across diverse anxiety disorders (Crombie and O'Connor, 2024; Solmi et al., 2025). Exercise interventions offer notable advantages, including low cost, generally few adverse effects, and ready scalability in community settings, features that may render them particularly attractive for SAD patients who tend to avoid conventional medical environments. Recent reviews have further emphasised that exercise may improve mental health through neurobiological and psychosocial mechanisms, including modulation of stress pathways, enhancement of neuroplasticity, and promotion of emotional resilience (Vancampfort et al., 2025). Because much of this evidence derives from general or mixed anxiety samples, however, it cannot be uncritically extrapolated to SAD, whose defining feature is fear of social evaluation rather than diffuse anxiety. Exercise may nonetheless be especially pertinent to SAD: group and outdoor formats provide graded exposure to social situations, while gains in self-efficacy, body image, and physical competence target core maintaining factors of social-evaluative fear, extending beyond exercise’s general anxiolytic effects.

**Figure 1:**
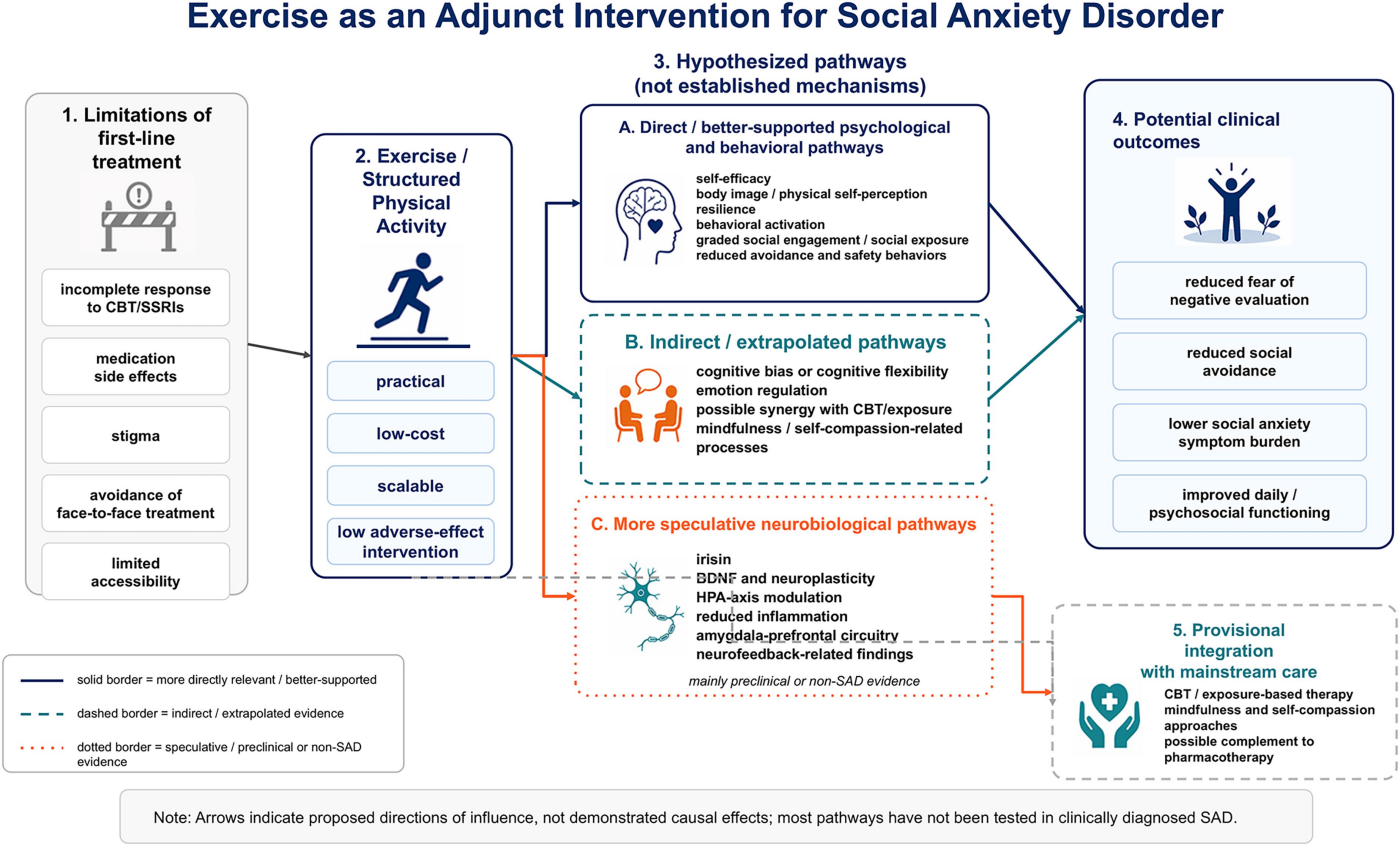
Proposed hypothesized pathways linking exercise to improvements in social anxiety disorder. Exercise or structured physical activity may serve as an adjunctive intervention for social anxiety disorder (SAD) in the context of incomplete response, tolerability issues, stigma, avoidance of face-to-face treatment, and limited access to first-line care. The proposed pathways are explicitly graded according to evidentiary proximity rather than presented as established mechanisms. Solid borders indicate comparatively better-supported and more directly relevant psychological and behavioral pathways, including self-efficacy, body image or physical self-perception, resilience, behavioral activation, graded social engagement or social exposure, and reduced avoidance and safety behaviors. Dashed borders indicate indirect or extrapolated pathways, including cognitive bias or cognitive flexibility, emotion regulation, possible synergy with CBT or exposure-based therapy, and mindfulness- or self-compassion-related processes. Dotted borders indicate more speculative neurobiological pathways that rely mainly on preclinical, transdiagnostic, or non-SAD evidence, including irisin, BDNF-related neuroplasticity, HPA-axis modulation, reduced inflammation, amygdala–prefrontal circuitry, and neurofeedback-related findings. Arrows represent proposed directions of influence rather than demonstrated causal effects, and most pathways have not been directly tested in clinically diagnosed SAD. The integration of exercise with mainstream care, including CBT/exposure-based therapy, mindfulness- and self-compassion-based approaches, and pharmacotherapy, should therefore be interpreted as provisional. BDNF, brain-derived neurotrophic factor; CBT, cognitive-behavioral therapy; HPA, hypothalamic–pituitary–adrenal; SAD, social anxiety disorder.

This mini-review synthesizes the clinical evidence for exercise interventions in social anxiety, examines their relationship with mainstream therapies, explores putative mechanisms, identifies current research gaps, and outlines future directions, intending to inform integrative management strategies for SAD (Figure 1). Accordingly, this review is organized around one primary question: Does exercise or structured physical activity have a role as an adjunctive intervention for clinically diagnosed SAD, and, if so, through what pathways? and four subsidiary questions: (i) what direct evidence exists for exercise interventions in clinically diagnosed SAD; (ii) what can be inferred, more cautiously, from indirect evidence in subclinical, analogue, or transdiagnostic samples; (iii) how exercise might be integrated with established psychological and pharmacological treatments, particularly CBT and exposure; and (iv) which psychological, physiological, and behavioral mechanisms have been proposed, and how well supported each is. To keep these distinctions explicit, evidence drawn from subclinical or non-SAD populations and from mechanistic or special-population studies is presented as indirect and labelled accordingly, and the speculative future directions are demarcated from the current evidence base. The principal empirical studies, together with their evidence proximity to exercise for SAD, are catalogued in Table 1.

**Table 1:** Summary of the principal studies discussed in this mini-review.

Author (Year) [ref]	Design	Population / Diagnostic status	N	Intervention/Exposure	Comparator	Main social-anxiety outcome	Follow-up	Main finding	Key limitation	Evidence proximity
Zika and Becker (2021)	Systematic review and 3 meta-analyses	Clinical and non-clinical social anxiety (mixed)	Multiple	Physical activity/exercise	Inactive / control conditions	Social anxiety symptoms	Varies by study	Longitudinal exercise reduced social anxiety; more active individuals reported milder symptoms	Few clinical-SAD trials; heterogeneous designs	Direct (mixed; partly subclinical)
Solmi et al. (2025)	Umbrella review	Various anxiety disorders incl. Social phobia (not SAD-specific)	Multiple	Moderate-to-vigorous aerobic exercise	Varied comparators	Core anxiety symptoms (transdiagnostic)	Varies	Aerobic exercise an effective transdiagnostic intervention; medium effects	Transdiagnostic; not SAD-specific	Indirect (transdiagnostic)
Cheng et al. (2025)	Cross-sectional	Chinese university students (non-clinical)	Students	Self-reported physical activity (type, intensity, duration)	None (observational)	Social anxiety (self-report)	None	Higher activity (esp. ≥1 h moderate-vigorous, 1–2×/week) linked to lower social anxiety	Cross-sectional; non-clinical; self-report	Indirect (observational, subclinical)
Fu et al. (2025)	Randomized controlled trial	Female college students (non-clinical)	Students	Therapeutic group exercise games (Yalom principles)	Conventional group aerobic exercise	Social interaction anxiety; fear of negative evaluation	Post-intervention	Group games superior to aerobic exercise; gender-specific efficacy	Small, non-clinical; single-sex; short follow-up	Partly direct (RCT, subclinical)
Gürer et al. (2024)	Intervention study	Adolescents (non-clinical)	Adolescents	Rock climbing	Baseline /comparison	Separation & generalized anxiety (not SAD-specific)	Short-term	Climbing associated with lower anxiety and improved well-being	Non-clinical; SAD not the primary outcome	Indirect (adjacent anxiety)
Özcan et al. (2025)	Intervention (12 weeks)	Children 12–14 y (non-clinical)	Children	Basic soccer training	Non-training comparison	Social physique anxiety	Post-intervention	No significant short-term effect on social physique anxiety	Small, non-clinical paediatric; short-term	Indirect (null; subclinical)
Li et al. (2024)	Cross-sectional (serial mediation)	Chinese college students (non-clinical)	Students	Self-reported physical activity	None	Social anxiety (self-report)	None	Activity linked to lower social anxiety via resilience and sleep	Cross-sectional; mediation, not causal	Indirect (observational, mechanism)
Wu et al. (2025)	Cross-sectional (serial mediation)	Adolescents (non-clinical)	Adolescents	Self-reported physical exercise	None	Social anxiety (self-report)	None	Exercise reduced social anxiety via sports self-efficacy and reduced expressive suppression	Cross-sectional; self-report	Indirect (observational, mechanism)
Xing et al. (2026)	Cross-sectional (mediation)	Junior-high students (non-clinical)	Students	Physical exercise	None	Social anxiety (self-report)	None	Exercise alleviated social anxiety partly through improved body image	Cross-sectional; adolescents; self-report	Indirect (observational, mechanism)
Weisman and Rodebaugh (2020)	Randomized experiment	Undergraduates with public-speaking anxiety (analogue)	Undergrads	Moderate-intensity exercise after exposure	Seated rest	Public speaking / social anxiety during exposure	Post-exposure	Exercise gave no advantage over rest; higher perceived exertion predicted worse outcomes	Analogue sample; single session; null result	Direct test of exercise (analogue)
Caudle et al. (2023)	Randomized adjunct trial	Adults with social anxiety (elevated/clinical)	Adults	Working-memory training adjunct to exposure	Exposure without training	Fear outcomes; neural activation	Post-intervention	Adjunctive cognitive training enhanced fear and neural outcomes	Cognitive training, not exercise; small	Indirect (adjunctive, non-exercise)
Rubin et al. (2022)	Pilot randomized controlled trial	Adults with SAD (clinical)	Adults	VR exposure therapy + attention-guidance training	VR exposure alone	Social anxiety symptoms	Post-intervention	Guiding attention away from social threat may reduce social anxiety	Pilot; small; not exercise	Indirect (adjunctive, non-exercise)
Teale Sapach and Carleton (2023)	Preliminary randomized controlled trial	Adults with SAD (clinical)	Adults	Self-compassion training	Relaxation training	Social anxiety; self-compassion	Post-intervention	Comparable symptom reduction; greater gains in self-compassion	Preliminary; not exercise	Indirect (comparator, non-exercise)

Studies are grouped by their proximity to the central question. “Direct” denotes a study that tested exercise or physical activity in clinically diagnosed SAD; “partly direct” denotes an exercise intervention in a subclinical or analogue sample; and “indirect” denotes observational, transdiagnostic, mechanistic, or non-exercise adjunctive studies that inform, but do not directly establish, the role of exercise in SAD. Abbreviations: RCT, randomized controlled trial; SAD, social anxiety disorder; VR, virtual reality.

## 2 Literature search and study selection

This narrative mini-review does not apply the formal protocol or reproducibility standards of a systematic review; instead, it offers a transparent account of how the cited literature was identified and selected. We searched PubMed/MEDLINE, Web of Science, Scopus, and PsycINFO, supplemented by Google Scholar for forward-citation tracking, from database inception to March 2026 (most recent search: March 2026). Search strings combined three concept blocks with Boolean operators: (i) the condition (“social anxiety disorder,” “social phobia,” “social anxiety,” “fear of negative evaluation,” “performance anxiety”); (ii) the exposure or intervention (“exercise,” “physical activity,” “aerobic exercise,” “resistance training,” “sport,” “climbing,” “group exercise,” “exergaming”); and (iii) selected mechanistic or contextual terms (“self-efficacy,” “body image,” “resilience,” “BDNF,” “irisin,” “HPA axis,” “cognitive bias,” “autism spectrum disorder,” “music performance anxiety”). Reference lists of key articles and pertinent reviews were hand-searched (snowballing) to identify additional sources, and only peer-reviewed articles published in English were considered.

Because the central question concerns exercise for clinically diagnosed SAD, an area in which adequately powered trials remain scarce, studies were prioritized, in descending order of relevance, as follows: (1) randomized controlled trials and other intervention studies of exercise or physical activity in clinically diagnosed SAD; (2) intervention and observational studies in subclinical or analogue social anxiety; (3) transdiagnostic exercise studies that explicitly reported social-anxiety or social-phobia outcomes; and (4) studies addressing the integration of exercise with cognitive-behavioral therapy (CBT) or exposure. Studies that did not directly address exercise, including mechanistic (e.g., irisin/BDNF), neuroimaging, pharmacotherapy design, and special-population (autism spectrum disorder, music performance anxiety) work, were included selectively, and only to illustrate plausible pathways, comparators, or translational context, rather than as direct evidence that exercise reduces social anxiety. Throughout the review, we therefore signpost the type and proximity of the evidence underlying each claim, distinguishing clinically diagnosed SAD from subclinical or analogue samples, exercise interventions from general psychological interventions, randomized trials from observational or cross-sectional designs, and tested clinical effects from hypothesized mechanisms. Given the narrative format, this account is intended to render our selection transparent rather than exhaustive, and some relevant studies may not have been captured.

## 3 Clinical evidence for exercise interventions in social anxiety

Table 1 summarizes the principal studies discussed in this review and indicates, for each, its design, the population and diagnostic status, and, critically, whether it provides direct or only indirect evidence on exercise for clinically diagnosed SAD. Cross-sectional and longitudinal investigations provide foundational evidence for an inverse association between physical activity and social anxiety. A study of Chinese university students reported a significant negative correlation between physical activity level and social anxiety, with exercise duration exerting the most pronounced effect on symptom reduction (Cheng et al., 2025). A systematic review and meta-analysis further demonstrated that longitudinal exercise interventions produced significant reductions in social anxiety symptoms, while cross-sectional evidence indicated that more physically active individuals reported milder social anxiety (Zika and Becker, 2021). Collectively, these findings suggest that enhanced physical activity may represent a viable avenue for both prevention and alleviation of social anxiety. Because much of this evidence is cross-sectional or observational, it establishes association rather than causation; moreover, the predominance of non-clinical and subclinical samples means these findings may not generalise directly to patients with clinically diagnosed SAD.

Randomized controlled trials (RCTs) have corroborated the efficacy of exercise-based approaches. A large umbrella review concluded that moderate-to-vigorous aerobic exercise functions as an effective transdiagnostic intervention, producing medium-sized effects on core symptoms of various anxiety disorders, including social phobia (Solmi et al., 2025). Specific exercise modalities have likewise shown encouraging results. For example, climbing has been associated with lower separation and generalized anxiety symptoms in adolescents (Gürer et al., 2024). Group-based exercise, particularly therapeutic group exercise games incorporating Yalomian therapeutic principles, demonstrated superior efficacy to conventional group aerobic exercise in reducing social interaction anxiety and fear of negative evaluation among female college students (Fu et al., 2025). These findings suggest that diverse exercise formats may confer benefit, with group-based formats offering additional opportunities for social exposure. It should be noted, however, that several of these modality-specific trials, including the climbing and group-exercise studies, were conducted in small, predominantly non-clinical samples, which limits their external validity and warrants cautious interpretation.

Regarding dose–response relationships, preliminary evidence indicates that moderate-to-vigorous exercise of at least 1 h per session, performed one to two or more times weekly, is associated with the lowest levels of social anxiety in university populations (Cheng et al., 2025). However, a 12-week soccer training program in children (a small, non-clinical paediatric sample) failed to produce significant short-term effects on social physique anxiety (Özcan et al., 2025), suggesting that outcomes are modulated by program design, population characteristics, and intervention duration. The benefits of exercise may further extend beyond anxiety itself: exercise has been shown to indirectly ameliorate comorbid depressive symptoms (Hagberg et al., 2023) and problematic smartphone use (Zhu et al., 2025; Wang et al., 2025), potentially through enhancement of psychological resilience (Li et al., 2024) and self-control (Wang et al., 2025).

## 4 Exercise in relation to mainstream psychological and pharmacological therapies

The interplay between exercise and traditional CBT and cognitive therapy (CT) constitutes an active research area. Studies examining working memory training as an adjunct to exposure therapy have demonstrated enhanced learning during exposure and altered neural activation patterns relevant to emotional processing (Caudle et al., 2023), providing a neurobiological rationale for the hypothesis that exercise-induced cognitive improvements may augment CBT efficacy. Although direct head-to-head comparisons between exercise and CBT remain scarce, a feasibility study of cognitive therapy based on the Clark and Wells model (CT-SAD-A) in adolescents demonstrated promising outcomes within routine child and adolescent mental health services, albeit with implementation challenges (Creswell et al., 2021). These observations suggest that structured psychotherapy and exercise likely address complementary dimensions, warranting further investigation of optimal integration strategies.

Evidence regarding exercise as an augmentation strategy for exposure therapy is mixed. One investigation hypothesized that exercise would potentiate extinction learning during exposure, yet found that moderate-intensity exercise performed immediately after exposure offered no advantage over seated rest in undergraduates with public speaking anxiety; participants reporting higher perceived exertion actually showed poorer anxiety outcomes (Weisman and Rodebaugh, 2020). Conversely, a pilot study combining virtual reality exposure therapy (VRET) with attention guidance training (AGT) indicated that modifying visual attention patterns toward social threats may reduce social anxiety, although larger samples are required (Rubin et al., 2022). These findings suggest that the manner in which exercise is integrated with exposure, including timing and modality, requires careful calibration.

Direct comparisons with pharmacotherapy represent a methodological frontier. The TAME trial, a non-inferiority RCT comparing mindfulness-based stress reduction with escitalopram for anxiety disorders (Hoge et al., 2020), offers a methodological template for head-to-head exercise–medication comparisons, though no large-scale RCT has yet directly compared exercise with first-line anxiolytics, an essential gap for future research. Because TAME compared mindfulness with escitalopram rather than testing exercise, it informs trial design only and cannot be read as evidence that exercise matches medication; at present, the evidence is insufficient to position exercise as a replacement for first-line CBT or SSRIs, and claims of equivalence would be premature.

Exercise exhibits natural synergy with “third-wave” cognitive-behavioral approaches such as mindfulness and self-compassion training. Within the transdiagnostic Unified Protocol, improvements in mindfulness skills correlate with anxiety symptom reduction, particularly among patients with generalized anxiety disorder (Woods et al., 2020). In SAD specifically, self-compassion training demonstrated efficacy comparable to relaxation training for symptom reduction, with superior gains in self-compassion itself (Teale Sapach and Carleton, 2023). Low-intensity mindfulness–CBT hybrid interventions also effectively reduced fear of negative evaluation and depressive symptoms in non-clinical samples (Noda et al., 2024). Because these approaches emphasize acceptance and awareness of internal experience, they may synergize with the capacity of exercise to foster mind–body integration.

## 5 Mechanisms underlying exercise-induced improvements in social anxiety

The mechanisms by which exercise ameliorates social anxiety are multifaceted, encompassing interacting psychological, physiological, and behavioral pathways. Because almost all of these mechanisms have been investigated outside clinically diagnosed SAD, they should be read as proposed rather than established pathways. We indicate the evidentiary status of each below and, in Figure 1, grade them accordingly: psychological and behavioral pathways (e.g., self-efficacy, body image, resilience, behavioral activation, and graded social engagement) are comparatively better supported and directly relevant; physiological and molecular pathways (e.g., irisin, BDNF, and HPA-axis modulation) rest largely on preclinical or non-SAD evidence and remain speculative in this context.

Psychologically, exercise enhances self-efficacy and psychological resilience. Physical training strengthens general self-efficacy and self-esteem, thereby attenuating feelings of inferiority (Liu, 2022), and psychological resilience mediates the relationship between physical activity and social anxiety (Li et al., 2024; Feng et al., 2024). Exercise additionally improves body image, which is intimately linked to social anxiety, with self-compassion operating as a mediator (Gao et al., 2023); among adolescents, body image enhancement represents a key pathway through which exercise reduces social anxiety (Xing et al., 2026). Exercise may further modulate cognitive biases: socially anxious individuals exhibit attentional (Liu et al., 2024; Günther et al., 2021) and interpretation biases (Prieto-Fidalgo and Calvete, 2024) toward threatening information. Although dedicated cognitive bias modification techniques (e.g., attention bias modification) directly target and alleviate these biases (Liu et al., 2024; Fadardi et al., 2023), whether regular exercise indirectly corrects them by enhancing cognitive flexibility and executive function remains to be fully elucidated.

Physiologically, exercise-induced irisin release represents an emerging focus. Irisin, a myokine released during physical activity, traverses the blood–brain barrier and upregulates brain-derived neurotrophic factor (BDNF), thereby enhancing neuroplasticity, suppressing neuroinflammation, and modulating hypothalamic–pituitary–adrenal (HPA) axis activity (Tatode et al., 2026). These actions align closely with the neurobiology of anxiety, providing a molecular rationale for the anxiolytic effects of exercise. It should be stressed, however, that this pathway rests largely on preclinical and indirect evidence, and a direct causal link between exercise-induced irisin and clinical anxiety reduction in humans has not been demonstrated. Neuroimaging research further demonstrates that fMRI-based neurofeedback can train adolescents to modulate prefrontal–amygdala connectivity, thereby altering social avoidance behavior (Lipp and Cohen Kadosh, 2020; Lisk et al., 2020), suggesting that exercise may likewise strengthen emotion regulation networks. Several additional physiological and social pathways may also contribute, including exercise-related improvements in sleep quality, enhanced autonomic (parasympathetic) regulation, modulation of systemic inflammation, and the social reinforcement afforded by group-based activity, although these mechanisms remain to be tested specifically in SAD (Li et al., 2024; MacKay et al., 2024).

Behaviorally, exercise offers a direct route to therapeutic benefit. The maintenance of social anxiety is tightly linked to frequent use of safety behaviors and social avoidance (Prieto-Fidalgo and Calvete, 2024; Rubin et al., 2020). Exercise itself constitutes behavioral activation, directly increasing both physical activity levels and social engagement. Following successful exposure therapy, patients exhibit greater daily physical activity and more frequent interactions with strangers (Heinig et al., 2024). Group exercise, in particular, provides a structured and predictable social exposure environment in which individuals can rehearse social skills at lower threat thresholds and progressively diminish avoidance (Reichenberger et al., 2022). Furthermore, enhanced exercise self-efficacy reduces expressive suppression, thereby mitigating social anxiety (Wu et al., 2025).

## 6 Applications in special populations and contexts

### 6.1 Children and adolescents with social anxiety

Early intervention is critical in children and adolescents. For behaviorally inhibited preschoolers, the parent-focused “Cool Little Kids” program and the integrated parent–child “Turtle Program” both effectively reduce anxiety, yet the latter produces superior gains in parenting behavior, particularly for parents with social anxiety themselves (Chronis-Tuscano et al., 2022). For diagnosed preschool SAD, internet-delivered family behavioral interventions (e.g., iCALM) show promising initial efficacy (Comer et al., 2021). In school settings, structured group interventions combining play, modeling, reinforcement, social skills training, and psychoeducation effectively mitigate the negative consequences of shyness (Cordier et al., 2021). These programs provide templates for integrating exercise with social–emotional learning in routine school activities.

### 6.2 Autism spectrum disorder with comorbid social anxiety

Individuals with autism spectrum disorder (ASD) frequently present with severe comorbid social anxiety. Beyond well-established social skills group training, such as PEERS®, which simultaneously improves social skills and anxiety symptoms (Parenteau et al., 2024; Afsharnejad et al., 2022), exercise has attracted particular attention for its capacity to induce irisin release; preclinical evidence indicates that irisin modulates ASD-relevant neurobiological pathways and ameliorates social and anxious behaviors in ASD animal models (Tatode et al., 2026). Developing combined exercise–social interventions (e.g., group exercise games), therefore, represents a promising avenue for ASD with comorbid social anxiety. This rationale, however, rests largely on animal models and on a population whose social difficulties differ in origin from DSM-defined SAD; it should therefore be regarded as hypothesis-generating. Evidence from ASD samples cannot be transferred directly to clinically diagnosed SAD, and combined exercise–social programs in this group have not yet been tested.

### 6.3 Performance-related anxiety

For context-specific anxiety, such as music performance anxiety (MPA), conceptualized as a subtype of social anxiety (although MPA overlaps with, but is not synonymous with, DSM-defined SAD) (Gómez-López and Sánchez-Cabrero, 2024), integration of exercise with psychological training appears valuable. Acceptance and commitment therapy (ACT) group interventions effectively enhance psychological flexibility and reduce MPA in young singers, with durable effects (Clarke et al., 2020). ACT coaching for adolescent singers helps reframe performance anxiety cognitions by shifting attentional focus from audience-pleasing to values-based engagement (Paul et al., 2024). Collective prevention programs (e.g., ConfiDance) likewise produce significant and sustained reductions in performance anxiety among music students (Gómez-López and Sánchez-Cabrero, 2024), and VRET provides innovative tools for simulating controlled performance exposures (Bellinger et al., 2023; Arif et al., 2023). Incorporating regular physical training into these frameworks could yield additional benefits through enhanced physiological tolerance, improved body image, and greater stress recovery capacity. Findings from MPA and other performance-anxiety contexts nevertheless have only limited and indirect bearing on DSM-defined SAD. They are included here as a related, illustrative context rather than as direct evidence for exercise in clinically diagnosed SAD.

## 7 Current gaps and limitations

Despite accumulating evidence, several important gaps remain. First, large-scale, high-quality RCTs in patients with clinically diagnosed SAD are conspicuously scarce (Crombie and O'Connor, 2024; Bandelow and Wedekind, 2022). Many existing studies feature small samples drawn from non-clinical or subclinical populations (e.g., students with elevated trait anxiety), limiting generalizability (Domschke et al., 2024b; Zika and Becker, 2021; Clarke et al., 2020; Bandelow and Wedekind, 2022). Second, long-term follow-up data are markedly insufficient; most studies assess outcomes only at post-intervention or short-term follow-up (1–3 months), leaving unresolved the durability of gains and whether sustained exercise participation is required to maintain them (Crombie and O'Connor, 2024).

Third, mechanistic research remains at an early stage. Although multiple pathways have been proposed, including psychological resilience, neuroplasticity, and irisin signaling (Li et al., 2024; Tatode et al., 2026), most evidence is indirect and lacks causal-chain studies that directly link exercise-induced changes in specific biomarkers (e.g., central irisin levels, targeted neural circuit connectivity) to anxiety reduction in humans (Tatode et al., 2026; MacKay et al., 2024). Fourth, personalization of intervention protocols is limited. Sex may moderate treatment effects (Crombie and O'Connor, 2024; Fu et al., 2025), and baseline anxiety severity, comorbidities such as depression, treatment-resistance history (Domschke et al., 2024b), as well as exercise preferences and expectations, are likely to influence engagement and benefit; yet guidance for precision exercise prescription based on these factors is currently lacking. Finally, methodological limitations constrain ecological validity. Many internet-based psychological intervention studies recruit predominantly highly educated samples, and remote diagnostic and therapist qualifications vary considerably, restricting external validity (Bandelow and Wedekind, 2022). Control conditions are frequently waitlist designs rather than active comparators (e.g., low-intensity exercise or health education), making it difficult to exclude non-specific effects such as attention and expectancy (Frommelt et al., 2023; Clark et al., 2024).

## 8 Future directions

To advance the field, several priorities merit emphasis. First, mechanism-driven precision-prescription research should move beyond the question of whether exercise is effective to identify which modalities, doses, and individual profiles yield maximal benefit. Factorial trials incorporating genetic, neuroendocrine, and baseline cognitive–behavioral data are needed to delineate active components and optimal parameters (Solmi et al., 2025; Cheng et al., 2025).

Second, deep integration of exercise with digital mental health technologies warrants exploration. Virtual reality has already been applied to exposure therapy (Rubin et al., 2022; Arif et al., 2023) and social skills training (Reichenberger et al., 2022) for social anxiety; future work could embed exercise biofeedback (e.g., heart rate, movement metrics) into VR environments to create immersive, gamified “exergaming” protocols, for example by pairing climbing (Gürer et al., 2024) or group exercise (Fu et al., 2025) with VR social contexts to enhance motivation and efficacy. Such exergaming applications remain an exploratory concept that has yet to be empirically validated in SAD and should be regarded as a hypothesis-generating direction. Telehealth platforms could likewise incorporate personalized exercise modules to complement internet-based CBT (Bandelow and Wedekind, 2022).

Third, integrated protocols for specific subpopulations should be developed. For treatment-resistant SAD (Domschke et al., 2024b), synergies with pharmacological augmentation (e.g., SSRI combinations) or novel psychotherapies such as imagery rescripting merit investigation. For complex cases involving comorbid ASD (Tatode et al., 2026; Afsharnejad et al., 2022), depression (Hagberg et al., 2023), or substance use (Buckner et al., 2020), multi-component stepped-care models positioning exercise as a core behavioral activation strategy should be designed.

Fourth, interdisciplinary collaboration is essential. Integrating perspectives from exercise science, neuroscience, immunopsychiatry, and clinical psychology, for example, by using fMRI to monitor real-time changes in emotion regulation networks before and after exercise (Lipp and Cohen Kadosh, 2020; Lisk et al., 2020), and by measuring peripheral irisin and inflammatory markers (Tatode et al., 2026; Albantakis et al., 2021), will deepen mechanistic understanding and generate novel intervention targets.

Fifth, methodological rigor in clinical trials should be strengthened through the use of active control arms (e.g., relaxation training, health education), blinded outcome assessment by independent clinicians, multi-level outcome measures spanning symptomatology, cognitive biases, and real-world social functioning and physical activity (Heinig et al., 2024; Rezeppa et al., 2021), and, where feasible, follow-up durations of 1 year or longer to evaluate durability.

Finally, translation of evidence into clinical and community practice requires the development of evidence-based clinical guidelines or expert consensus statements on integrating exercise into SAD management, cross-training of mental health and exercise professionals, and implementation studies testing structured programs in community centers, schools, and workplaces to maximize accessibility and public health impact (Creswell et al., 2021; Cordier et al., 2021).

## 9 Conclusion and outlook

Taken together, the available evidence, most of which derives from subclinical, analogue, or transdiagnostic samples rather than from adequately powered trials in clinically diagnosed SAD, suggests that regular physical activity and structured exercise, being generally well tolerated, broadly accessible, and associated with few serious adverse effects in the populations studied to date, show preliminary but encouraging promise for reducing social anxiety symptoms and enhancing psychosocial functioning. Exercise may not only alleviate anxiety directly but also operate through multiple pathways, including enhancement of self-efficacy and psychological resilience, modulation of cognitive biases, and reduction of avoidance behaviors. Therefore, it appears to be a promising adjunctive intervention and may serve as an option for selected individuals who cannot access or tolerate standard treatments; current evidence, however, does not support replacing established first-line therapies such as CBT or SSRIs. It may be particularly well-suited to individuals who are wary of conventional treatments, who respond inadequately to them, or who prefer a holistic health-management strategy.

Nonetheless, the field remains at a developmental stage, with many critical questions unresolved. Rigorous, adequately powered RCTs with representative samples and extended follow-up are required to consolidate the evidence base, alongside interdisciplinary research elucidating the biopsychosocial mechanisms underlying the anxiolytic effects of exercise. Building on such foundations, the development of personalized, precision exercise prescriptions and optimal integration with emerging digital technologies and established therapeutic paradigms will constitute central tasks for the field.

The ultimate objective is to incorporate regular exercise scientifically and systematically as one evidence-based component within the broader, multimodal management of social anxiety disorder, a goal that remains contingent on the stronger, SAD-specific trial evidence outlined above. Realizing this goal will require sustained engagement not only from researchers but also from clinicians, public health policymakers, and community organizations, collectively expanding the range of recovery options that are diverse, effective, and accessible to individuals affected by social anxiety.
